# Therapeutic Effects of Procainamide on Endotoxin-Induced Rhabdomyolysis in Rats

**DOI:** 10.1371/journal.pone.0150319

**Published:** 2016-02-26

**Authors:** Chih-Chin Shih, Hiong-Ping Hii, Cheng-Ming Tsao, Shiu-Jen Chen, Shuk-Man Ka, Mei-Hui Liao, Chin-Chen Wu

**Affiliations:** 1 Graduate Institute of Medical Sciences, National Defense Medical Center, Taipei, R.O.C., Taiwan; 2 Department of Pharmacology, National Defense Medical Center, Taipei, R.O.C., Taiwan; 3 Department of Surgery, Chi Mei Medical Center, Tainan, R.O.C., Taiwan; 4 Department of Anesthesiology, Taipei Veterans General Hospital and National Yang-Ming University, Taipei, R.O.C., Taiwan; 5 Department of Physiology, National Defense Medical Center, Taipei, R.O.C., Taiwan; 6 Departments of Nursing, Kang-Ning Junior College of Medical Care and Management, Taipei, R.O.C., Taiwan; 7 Departments of Health Management for Elderly Society, Kang-Ning Junior College of Medical Care and Management, Taipei, R.O.C., Taiwan; 8 Graduate Institute of Aerospace and Undersea Medicine, National Defense Medical Center, Taipei, R.O.C., Taiwan; King Abdullah International Medical Research Center, SAUDI ARABIA

## Abstract

Overt systemic inflammatory response is a predisposing mechanism for infection-induced skeletal muscle damage and rhabdomyolysis. Aberrant DNA methylation plays a crucial role in the pathophysiology of excessive inflammatory response. The antiarrhythmic drug procainamide is a non-nucleoside inhibitor of DNA methyltransferase 1 (DNMT1) used to alleviate DNA hypermethylation. Therefore, we evaluated the effects of procainamide on the syndromes and complications of rhabdomyolysis rats induced by lipopolysaccharide (LPS). Rhabdomyolysis animal model was established by intravenous infusion of LPS (5 mg/kg) accompanied by procainamide therapy (50 mg/kg). During the experimental period, the changes of hemodynamics, muscle injury index, kidney function, blood gas, blood electrolytes, blood glucose, and plasma interleukin-6 (IL-6) levels were examined. Kidneys and lungs were exercised to analyze superoxide production, neutrophil infiltration, and DNMTs expression. The rats in this model showed similar clinical syndromes and complications of rhabdomyolysis including high levels of plasma creatine kinase, acute kidney injury, hyperkalemia, hypocalcemia, metabolic acidosis, hypotension, tachycardia, and hypoglycemia. The increases of lung DNMT1 expression and plasma IL-6 concentration were also observed in rhabdomyolysis animals induced by LPS. Treatment with procainamide not only inhibited the overexpression of DNMT1 but also diminished the overproduction of IL-6 in rhabdomyolysis rats. In addition, procainamide improved muscle damage, renal dysfunction, electrolytes disturbance, metabolic acidosis, hypotension, and hypoglycemia in the rats with rhabdomyolysis. Moreover, another DNMT inhibitor hydralazine mitigated hypoglycemia, muscle damage, and renal dysfunction in rhabdomyolysis rats. These findings reveal that therapeutic effects of procainamide could be based on the suppression of DNMT1 and pro-inflammatory cytokine in endotoxin-induced rhabdomyolysis.

## Introduction

Rhabdomyolysis is a fatal syndrome that results from severe muscle damage and the subsequent leakage of intramuscular contents into the circulation [1]. Acute kidney injury, electrolyte disturbance, and metabolic acidosis are the major clinical complications of rhabdomyolysis [2, 3]. The mortality and prognosis of rhabdomyolysis are dependent upon these associated comorbidities. Therefore, it is necessary to discover new medical therapy for further improvement of the complications in rhabdomyolysis.

There are many potential causes that destroy muscle tissues, leading to rhabdomyolysis. Although traumatic injury is the most common condition to induce rhabdomyolysis, additional causes including infections, toxins, exertion, medication, and hyperthermia can also initiate rhabdomyolysis [4]. Severe blood infection often induces systemic inflammatory responses throughout the body to destroy multiple organ systems and develop sepsis. Association between bacterial sepsis and rhabdomyolysis has been observed in past reports [5, 6]. Rhabdomyolysis in infectious or sepsis patients have higher incidence of complications and higher mortality [7, 8]. However, the mechanism on the development of rhabdomyolysis during bacterial sepsis has not been clarified. To deal with this problem, we established an endotoxin-induced rhabdomyolysis animal model by intravenous infusion of lipopolysaccharide (LPS). The rats in this model showed clinical manifestations of rhabdomyolysis ranging from the elevation of plasma creatine kinase (CK) levels to acute kidney injury, electrolyte imbalance, and metabolic acidosis.

Overproduction of pro-inflammatory mediators is a predisposing mechanism for infection-induced skeletal muscle damage [9]. The disruption of skeletal muscle integrity evokes the release of intracellular components and results in rhabdomyolysis. DNA methylation is an epigenetic mechanism and regulated by DNA methyltransferases (DNMTs) to modify the expression of multiple genes [10]. Pathogenic bacteria and its component are crucial mediators to alter DNA methylation in the host. Previous studies demonstrate that DNMT1 expression and DNA methylation were significantly increased after infection with uropathogenic *Escherichia coli* or exposure to LPS [11–14]. In addition, the changes of DNA methylation status have been associated with the regulation of inflammation. Elevation of cytokine levels in macrophages is triggered by homocysteine via DNA methylation enhancement [15]. Oscillatory shear stress augments DNA methylation to cause endothelial inflammation which can be diminished by DNMT inhibitor 5-aza-2′-deoxycytidine (5-aza-dC) or DNMT1 siRNA [16]. Treatment with 5-aza-dC also ameliorates macrophage inflammation, migration, and adhesion in atherosclerotic plaques [17]. Moreover, DNMT1 inhibitors can reduce the secretion of inflammatory cytokines in LPS-induced macrophages by diminishing suppressor of cytokine signaling 1 (SOCS1) hypermethylation [14]. These findings indicate that DNA methylation status plays an important role in the regulation of inflammation and skeletal muscle damage induced by bacterial toxic component.

Nucleoside and non-nucleoside analogues are two families of DNMT inhibitors used to alleviate DNA hypermethylation in diseases. Nucleoside inhibitors of DNMTs, such as 5-aza-dC, have been found to restore the hypermethylation genes and be potential chemotherapeutic agents [18]. However, the continuing therapeutic uses of these nucleoside analogs come with some serious side effects, such as myelotoxicity and mutation risk [19]. To overcome these concerns, non-nucleoside inhibitors of DNMTs have been developed. Of particular interest is procainamide, one of the class 1A antiarrhythmic drugs approved to treat a variety of atrial and ventricular arrhythmias. Procainamide is also a non-nucleoside specific inhibitor of DNMT1 that can manipulate aberrant DNA methylation [20, 21]. The expression of tumor suppressor genes silenced by DNA hypermethylation in cancer cells can be reactivated by procainamide [22, 23]. Taken together, microbial infection is a potential factor to cause skeletal muscle injury and rhabdomyolysis by augmenting systemic inflammatory responses. DNA hypermethylation plays a pathogenic role in these overt inflammatory responses in infectious diseases. Therefore, we examined the effects of procainamide on muscle injury, renal dysfunction, electrolytes imbalance, and metabolic acidosis in LPS-induced rhabdomyolysis animal model.

## Materials and Methods

### Ethics statement

All animal procedures were approved by the Institutional Animal Care and Use Committee of National Defense Medical Center (Taipei, R.O.C., Taiwan) (Permit Number: IACUC-12-131) and accomplished in adherence to the National Institutes of Health guidelines. Humane endpoints and euthanized animals prior to the end of the experiments were used in this study. We determined when the animals should be euthanized by the signs of weight loss, dyspnea, cyanosis, inappetence, extreme reluctance to stand, depression coupled with low body temperature, severe diarrhea, seizures, paralysis of one or more extremities. Overdose anesthetic (sodium pentobarbital) was the method of euthanasia in this study. The health of the rats were examined and monitored every 1 h, and there were no unexpected deaths in these cases. We used anesthesia (sodium pentobarbital) to reduce the distress and suffering of animals before any process that is potentially stressful or painful.

### Animals and experimental designs

Male Wistar rats (10–12 weeks old, 250–300 g) were obtained from BioLASCO Taiwan Co (Taipei, R.O.C., Taiwan). Rats were housed under a 12-h light/dark cycle and a controlled temperature (21 ± 2°C) with free access to water and food. Animals were anesthetized by sodium pentobarbital (50 mg/kg), and the right jugular vein was cannulated with polyethylene catheter for drug administration. The left carotid artery was cannulated with polyethylene catheter for blood sampling and connected to a pressure transducer (P23ID, Statham, Oxnard, CA, USA) to measure hemodynamic parameters, which were exhibited on a MacLab/4e poly-graph recorder (AD Instruments Pty Ltd., Castle Hill, Australia). After the cannulated rats recovered to normal condition overnight, they were randomly allocated into four groups as follows: (1) Control group (0.5 mL/kg of saline was given intravenously at time 0); (2) Control + Pro group (saline was given intravenously at time 0 and then 50 mg/kg procainamide was given by intravenous infusion over 30 min at 1 h); (3) LPS-induced rhabdomyolysis group (5 mg/kg *Escherichia coli* lipopolysaccharide (LPS) was given intravenously at time 0); (4) LPS-induced rhabdomyolysis + Pro group (LPS was given intravenously at time 0 and then 50 mg/kg procainamide was given by intravenous infusion over 30 min at 1 h). This study was performed and monitored for 6 h. During the experimental period, we examined changes in hemodynamics (i.e., mean arterial blood pressure [MAP] and heart rate [HR]), blood glucose, blood gas (i.e., HCO_3_^-^ and base excess [BE]), blood electrolytes (i.e., potassium and calcium), muscle injury index (i.e., creatine kinase [CK] and lactate dehydrogenase [LDH]), kidney function index (i.e., creatinine), and plasma interleukin-6 (IL-6) concentration. Each volume of blood drawn was replenished by the administration of an equal volume of saline. At 6 h after saline or LPS, kidneys were exercised to examine superoxide production and histological alternation. In addition, lungs were exercised to analyze the expression of DNMTs. In the separate experiments, effects of another DNMT inhibitor hydralazine [21, 23] on rhabdomyolysis rats were also performed and monitored for 6 h. Animals were divided into four groups: (1) Control group (0.5 mL/kg of saline was given intravenously at time 0); (2) Control + HYD group (saline was given intravenously at time 0 and then 0.3 mg/kg hydralazine was given by intravenous infusion over 15 min at 0.5 h); (3) LPS-induced rhabdomyolysis group (5 mg/kg LPS was given intravenously at time 0); (4) LPS-induced rhabdomyolysis + HYD group (LPS was given intravenously at time 0 and then 0.3 mg/kg hydralazine was given by intravenous infusion over 15 min at 0.5 h).

### Measurement of blood glucose

At baseline (i.e., time 0) and at 1, 2, 4, 6 h after saline or LPS, blood samples were taken to measure the glucose levels by a One Touch II blood glucose monitoring system (Lifescan, Milpitas, CA, USA).

### Measurement of blood gas and electrolytes

Arterial blood was used to analyze the levels of HCO_3_^-^, BE, potassium, and calcium by an arterial blood gas analyzer (AVL OPTI Critical Care Analyzer, AVL Scientific Corp., Roswell, GA, USA).

### Quantification of muscle injury and renal function

Blood samples were centrifuged at 16,000 *g* for 2 min to obtain the plasma for the measurement of biochemical variables. Forty microliters of plasma was used to evaluate muscle injury and renal function at baseline (i.e., time 0) and at 1, 2, 4, 6 h after saline or LPS. The increases in plasma levels of CK and LDH were regarded as biochemical indicators of muscle injury. The elevation in plasma level of creatinine was considered as biochemical indicator of kidney function. All of these biochemical variables were analyzed by Fuji DRI-CHEM 3030 (Fuji Photo Film, Tokyo, Japan).

### Measurement of plasma IL-6 levels

The plasma samples of rats obtained at 0 and 6 h were used to analyze IL-6 levels with an enzyme linked immunosorbent assay kit (R&D Systems, Minneapolis, MN, USA) according to the manufacturer’s protocol.

### Measurement of superoxide production in the kidney

The specimens of kidneys were harvested at 6 h and incubated with warmed (37°C), oxygenated (95% O_2_/5% CO_2_) Krebs-HEPES buffer for 10 min. These tissues were transferred to 96-well microplates containing Krebs-HEPES buffer with 1.25 mM lucigenin. The microplates were then placed into a microplate luminometer (Hidex Microplate Luminometer, Turku, Finland) to measure the counts. The specimens of kidneys were dried in the oven for 24 h, and the data were displayed as count per second per milligram of tissue dry weight.

### Histopathologic analysis

At the end of study, kidneys were obtained from animals and fixed in 10% formaldehyde. The fixed specimens were dehydrated in graded ethanol, embedded in paraffin, and stained with the hematoxylin and eosin. The infiltration of polymorphonuclear neutrophil (PMN) in the kidney was quantitatively studied as the severity of renal inflammation. The index of each tissue section was scored 0 (minimal) to 4 (maximal) by a pathologist in a blinded fashion.

### Western blot analysis

Lungs were obtained from rats at the end of experiment. Nuclear fraction was collected by using the CNM compartmental protein extraction kit (BioChain Inc., San Francisco, CA, USA) according to the manufacturer’s instruction. Twenty μg of protein was separated by 10% sodium dodecyl sulfate-polyacrylamide gel electrophoresis and transferred to nitrocellulose membrane. The membrane was incubated with primary antibody (DNMT1, 1:1000, purchased from Abcam, Cambridge, UK; DNMT3A, 1:300, purchased from Abcam, Cambridge, UK; DNMT3B, 1:1000, purchased from GeneTex, Irvine, CA, USA; Histone H3, 1:5000, purchased from GeneTex, Irvine, CA, USA) at 4°C for 24 h, and then incubated with horseradish peroxidase-conjugated donkey anti-goat IgG (Abcam, Cambridge, UK) or horseradish peroxidase-conjugated goat anti-rabbit IgG (Cell signaling Technology Inc, Danvers, MA, USA) at room temperature for 2 h. The protein levels were detected by the enhanced chemiluminescence Western blotting detection reagent (Thermo scientific, Rockford, IL, USA), and quantified by the ImageJ software version 1.46r (National Institutes of Health, Bethesda, MD, USA).

### Statistical analysis

All data are presented as mean ± standard error of the mean of *n* determinations, where *n* indicates the number of rats studied. The significance between groups was analyzed by using one-way analysis of variance followed by a multiple comparison test (Newman-Keuls test). A *p* value <0.05 was regarded as statistically significant.

## Results

### Effects of procainamide on muscle injury

**Fig 1:**
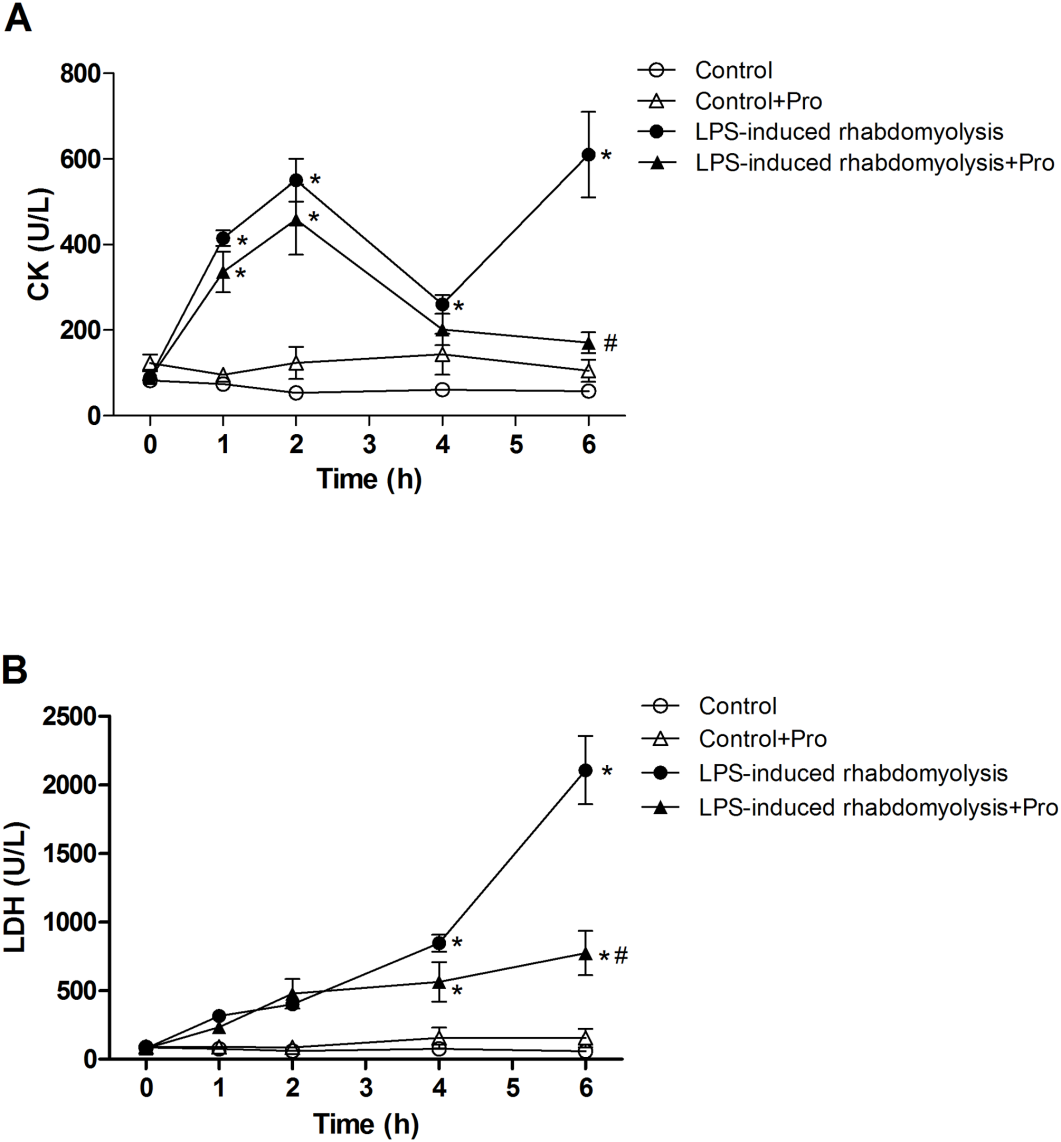
Effects of procainamide on (A) creatine kinase (CK) and (B) lactate dehydrogenase (LDH) in LPS-induced rhabdomyolysis rats. Depicted are the changes during the experimental period in different groups of animals that received vehicle at time 0 (Control, n = 6), received vehicle at time 0 and then received procainamide at 1 h (Control + Pro, n = 6), received LPS at time 0 (LPS-induced rhabdomyolysis, n = 8), and received LPS at time 0 and then received procainamide at 1 h (LPS-induced rhabdomyolysis + Pro, n = 8). All data are expressed as mean ± SEM. *P < 0.05, all versus Control rats; ^#^*P* < 0.05, with versus without procainamide in LPS-induced rhabdomyolysis rats.

The rats in the LPS-induced rhabdomyolysis group showed obvious increases in plasma levels of CK and LDH during the experimental period (Fig 1A and 1B). The treatment of rhabdomyolysis rats with procainamide significantly ameliorated the rises in plasma CK and LDH levels at 6 h after LPS (Fig 1A and 1B). However, no significant alterations in these biochemical markers were observed during the experimental period in the Control and Control + Pro groups.

### Effects of procainamide on electrolytes imbalance

**Fig 2:**
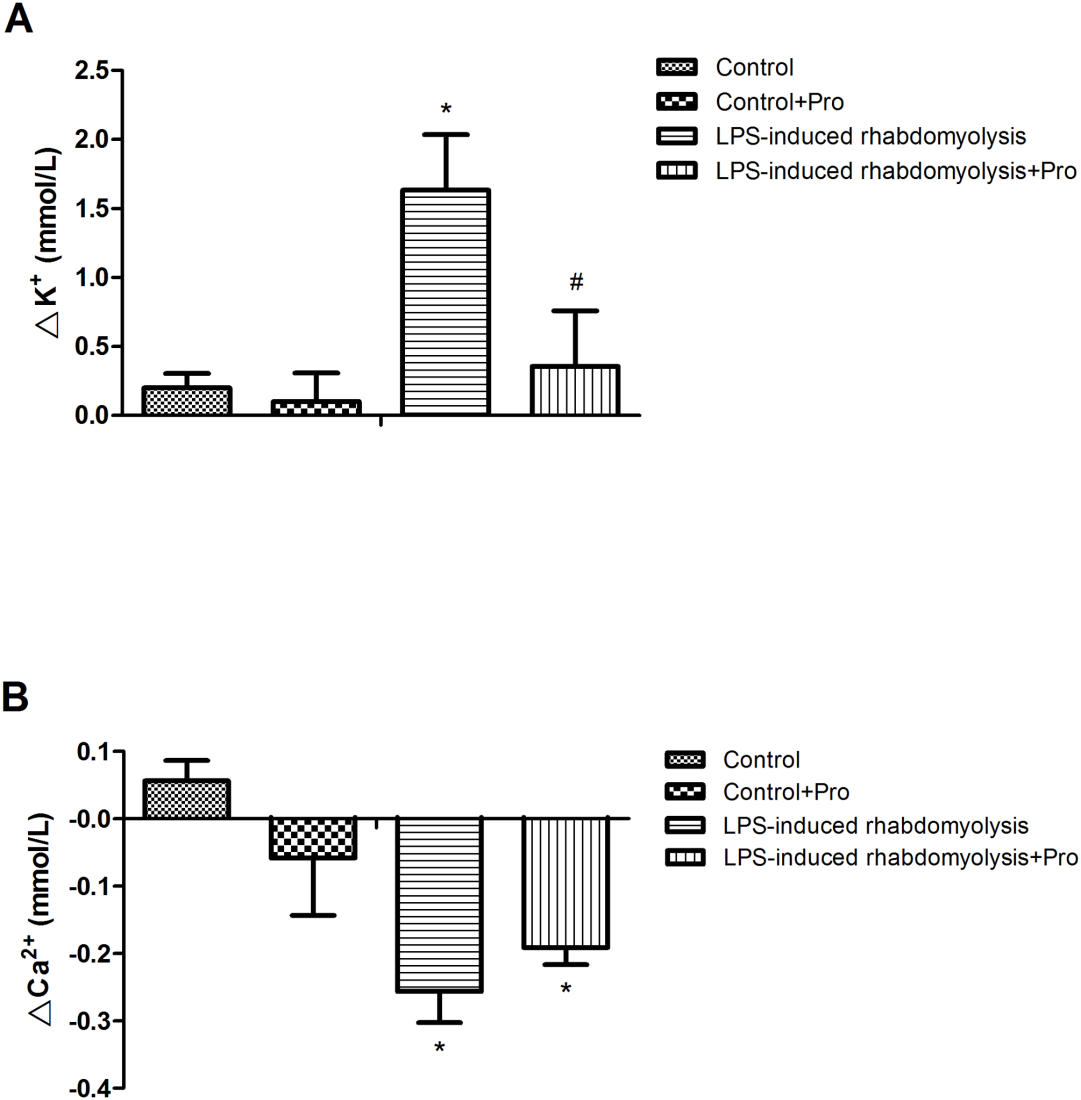
Effects of procainamide on (A) potassium and (B) calcium levels in the arterial blood of LPS-induced rhabdomyolysis rats. Depicted are the changes during the experimental period in different groups of animals that received vehicle at time 0 (Control, n = 5), received vehicle at time 0 and then received procainamide at 1 h (Control + Pro, n = 5), received LPS at time 0 (LPS-induced rhabdomyolysis, n = 6), and received LPS at time 0 and then received procainamide at 1 h (LPS-induced rhabdomyolysis + Pro, n = 6). The values of all groups are normalized at 0 h as zero. All data are obtained using the same rats in Fig 1 and expressed as mean ± SEM. *P < 0.05, all versus Control rats; ^#^*P* < 0.05, with versus without procainamide in LPS-induced rhabdomyolysis rats.

The animals in the LPS-induced rhabdomyolysis group exhibited an increase in potassium concentration (Fig 2A) and a decline in calcium concentration (Fig 2B) in the arterial blood at 6 h after LPS. Hyperkalemia caused by endotoxin was improved by procainamide administration, whereas hypocalcemia caused by endotoxin was not alleviated by procainamide administration. The treatment of Control rats with procainamide had no significant effect on the levels of potassium and calcium.

### Effects of procainamide on renal function

**Fig 3:**
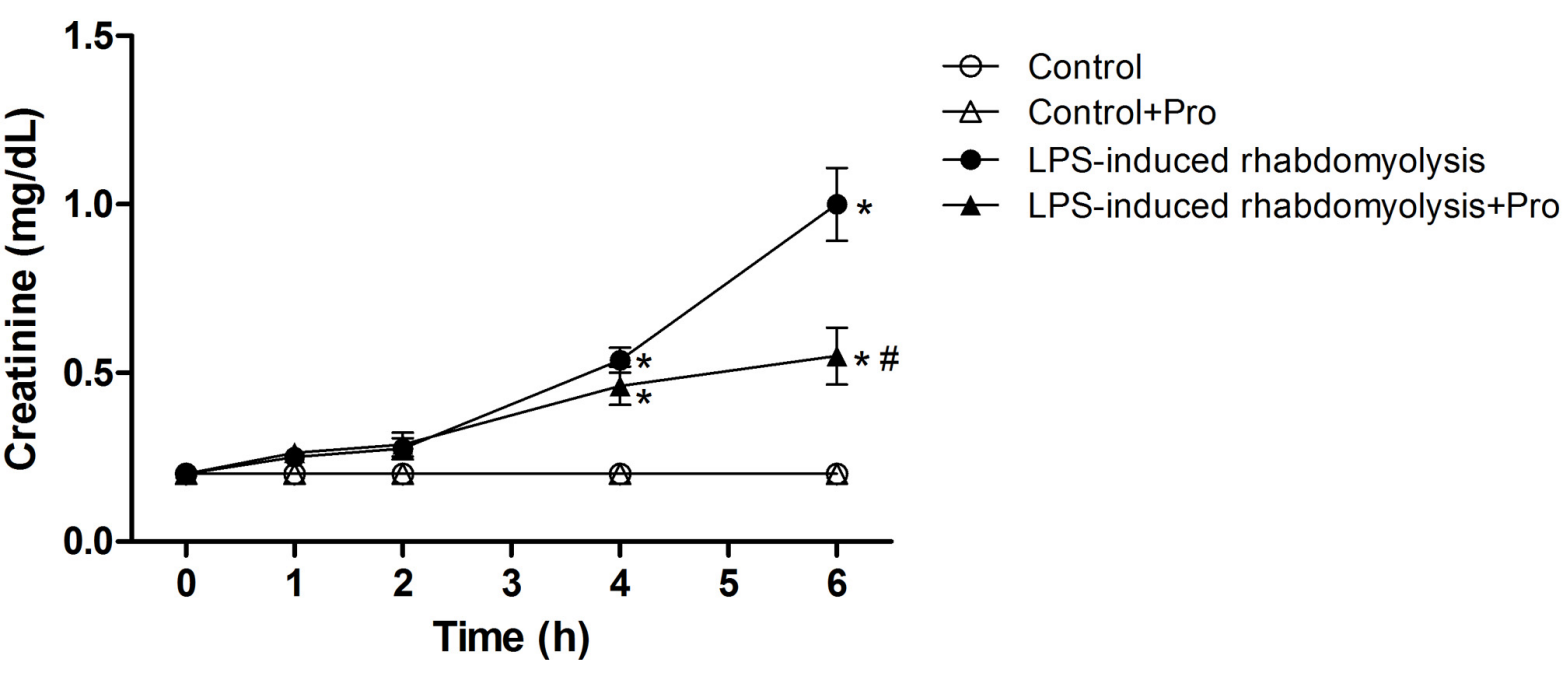
Effects of procainamide on creatinine in LPS-induced rhabdomyolysis rats. Depicted are the changes during the experimental period in different groups of animals that received vehicle at time 0 (Control, n = 6), received vehicle at time 0 and then received procainamide at 1 h (Control + Pro, n = 6), received LPS at time 0 (LPS-induced rhabdomyolysis, n = 8), and received LPS at time 0 and then received procainamide at 1 h (LPS-induced rhabdomyolysis + Pro, n = 8). All data are expressed as mean ± SEM. *P < 0.05, all versus Control rats; ^#^*P* < 0.05, with versus without procainamide in LPS-induced rhabdomyolysis rats.

No significant changes in plasma levels of creatinine were observed during the experimental period in the Control group treated with saline or procainamide (Fig 3). The LPS caused significant elevations in plasma levels of creatinine at 4 and 6 h (Fig 3). However, procainamide administration significantly attenuated the increase of plasma creatinine level at 6 h in the LPS-induced rhabdomyolysis group (Fig 3).

### Effects of procainamide on kidney superoxide production

**Fig 4:**
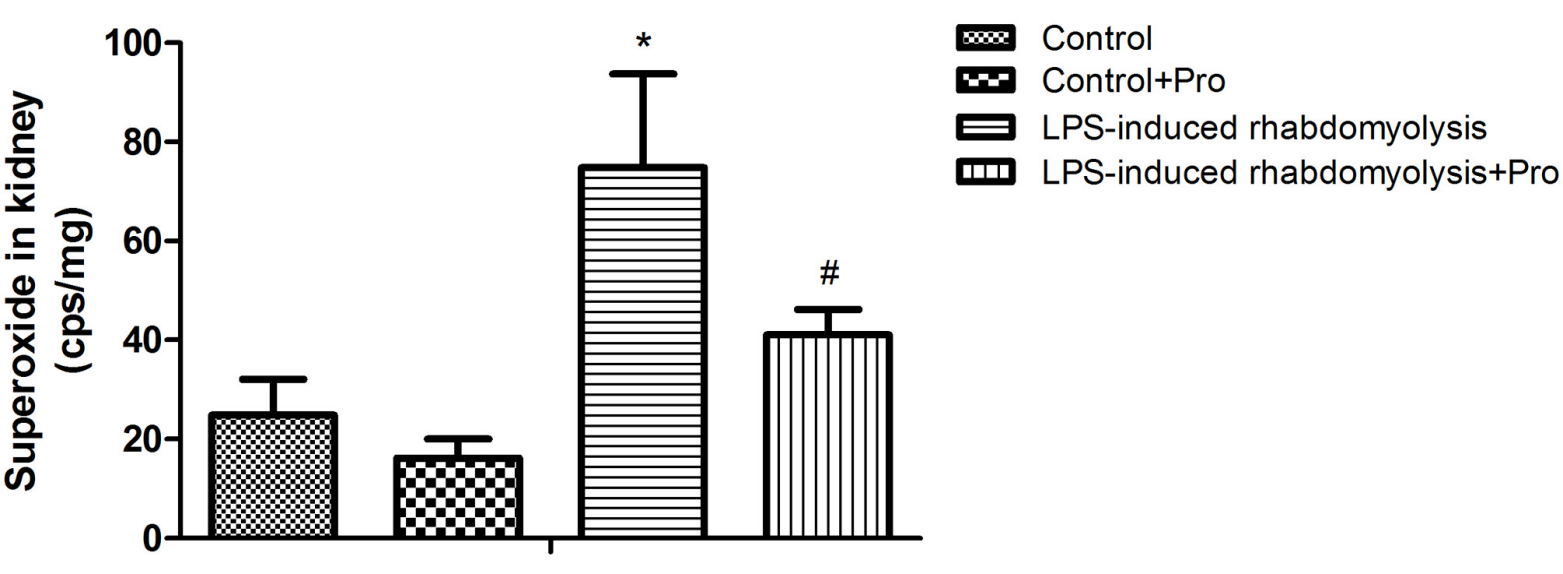
Effects of procainamide on superoxide levels in kidneys obtained from LPS-induced rhabdomyolysis rats. Depicted are changes at the end of experiments (at 6 h) in different groups of animals that received vehicle at time 0 (Control, n = 6), received vehicle at time 0 and then received procainamide at 1 h (Control + Pro, n = 6), received LPS at time 0 (LPS-induced rhabdomyolysis, n = 6), and received LPS at time 0 and then received procainamide at 1 h (LPS-induced rhabdomyolysis + Pro, n = 6). All data are obtained using the same rats in Fig 1 and expressed as mean ± SEM. *P < 0.05, all versus Control rats; ^#^*P* < 0.05, with versus without procainamide in LPS-induced rhabdomyolysis rats.

In the Control group, the basal production of superoxide was measurable in the kidney (Fig 4). The rats that received LPS had a significant increase in the superoxide level of kidney (Fig 4). The treatment of rhabdomyolysis rats with procainamide significantly inhibited superoxide production in the kidney (Fig 4). However, procainamide had no effects on the levels of kidney superoxide in Control animals (Fig 4).

### Effects of procainamide on metabolic acidosis

**Fig 5:**
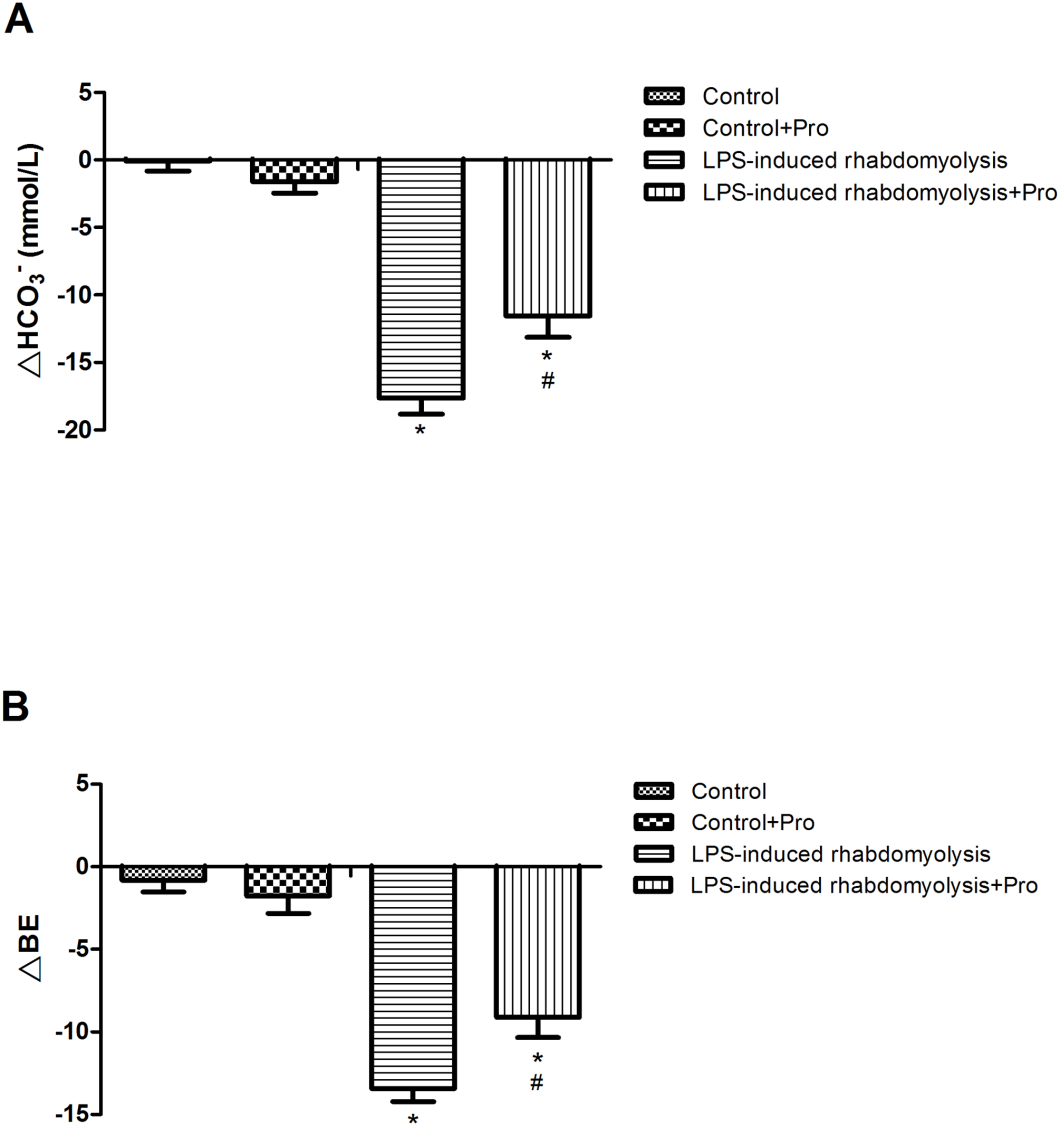
Effects of procainamide on (A) HCO3- and (B) base excess (BE) levels in the arterial blood of LPS-induced rhabdomyolysis rats. Depicted are the changes during the experimental period in different groups of animals that received vehicle at time 0 (Control, n = 5), received vehicle at time 0 and then received procainamide at 1 h (Control + Pro, n = 5), received LPS at time 0 (LPS-induced rhabdomyolysis, n = 6), and received LPS at time 0 and then received procainamide at 1 h (LPS-induced rhabdomyolysis + Pro, n = 6). The values of all groups are normalized at 0 h as zero. All data are obtained using the same rats in Fig 1 and expressed as mean ± SEM. *P < 0.05, all versus Control rats; ^#^*P* < 0.05, with versus without procainamide in LPS-induced rhabdomyolysis rats.

The rhabdomyolysis animals exhibited significant decreases in blood HCO_3_^-^ and BE levels at 6 h after LPS (Fig 5A and 5B). Administration of procainamide significantly improved metabolic acidosis in rhabdomyolysis rats (Fig 5A and 5B). However, there were no significant effects on blood levels of HCO_3_^-^ and BE after treatment of Control rats with procainamide (Fig 5A and 5B).

### Effects of procainamide on hemodynamic variables and blood glucose

The baseline values of MAP, HR, and blood glucose were not significantly different in all groups (Table 1). LPS led to a significant reduction in MAP and a significant rise in HR during the experimental period (Table 1). The treatment of rhabdomyolysis animals with procainamide significantly prevented hypotension at 4 and 6 h (Table 1). However, procainamide had no effects on tachycardia induced by endotoxin (Table 1). There was no significant difference in the values of MAP and HR between the LPS-induced rhabdomyolysis and LPS-induced rhabdomyolysis + Pro groups (Table 1).

**Table 1:** Effects of procainamide on hemodynamic parameters and blood glucose in LPS-induced rhabdomyolysis rats.

	0 h	1 h	2 h	4 h	6 h
**MAP (mmHg)**					
Control	121 ± 2	119 ± 3	117 ± 3	118 ± 2	116 ± 3
Control + Pro	115 ± 3	118 ± 5	119 ± 4	118 ± 4	114 ± 3
LPS-induced rhabdomyolysis	120 ± 2	72 ± 4*	111 ± 3	97 ± 4*	62 ± 3*
LPS-induced rhabdomyolysis + Pro	121 ± 2	76 ± 3*	116 ± 4	110 ± 4†	103 ± 5*†
**HR (beats/min)**					
Control	347 ± 9	332 ± 10	334 ± 6	338 ± 8	331 ± 10
Control + Pro	328 ± 23	314 ± 9	338 ± 19	358 ± 21	362 ± 15
LPS-induced rhabdomyolysis	353 ± 16	403 ± 31	519 ± 26*	570 ± 27*	531 ± 40*
LPS-induced rhabdomyolysis + Pro	346 ± 10	385 ± 15	456 ± 12*	531 ± 32*	597 ± 50*
**Blood glucose (mg/dL)**					
Control	101 ± 2	92 ± 3	91 ± 2	98 ± 3	97 ± 2
Control + Pro	99 ± 2	94 ± 3	101 ± 3	107 ± 3	101 ± 4
LPS-induced rhabdomyolysis	104 ± 2	220 ± 11*	103 ± 4	68 ± 4*	27 ± 3*
LPS-induced rhabdomyolysis + Pro	106 ± 5	215 ± 12*	94 ± 7	88 ± 4	70 ± 8*†

Shown are the changes in MAP, HR, and blood glucose during the experimental period in different groups of animals. Control, saline was given at time 0 (n = 6); Control + Pro, saline was given at time 0 and then 50 mg/kg procainamide was given at 1 h (n = 6); LPS-induced rhabdomyolysis, LPS was given at time 0 (n = 8); LPS-induced rhabdomyolysis + Pro, LPS was given at time 0 and then 50 mg/kg procainamide was given at 1 h (n = 8). All data are expressed as mean ± SEM.

*P < 0.05, all versus Control rats;

^†^*P* < 0.05, with versus without procainamide in LPS-induced rhabdomyolysis rats.

The rats in the LPS-induced rhabdomyolysis group showed significant decreases in the levels of blood glucose at 4 and 6 h after LPS (Table 1). The administration of rhabdomyolysis animals with procainamide significantly ameliorated hypoglycemia at 6 h (Table 1). However, there was no significant difference in the levels of blood glucose between the Control and Control + Pro groups (Table 1).

### Effects of procainamide on neutrophil infiltration in the kidney

**Fig 6:**
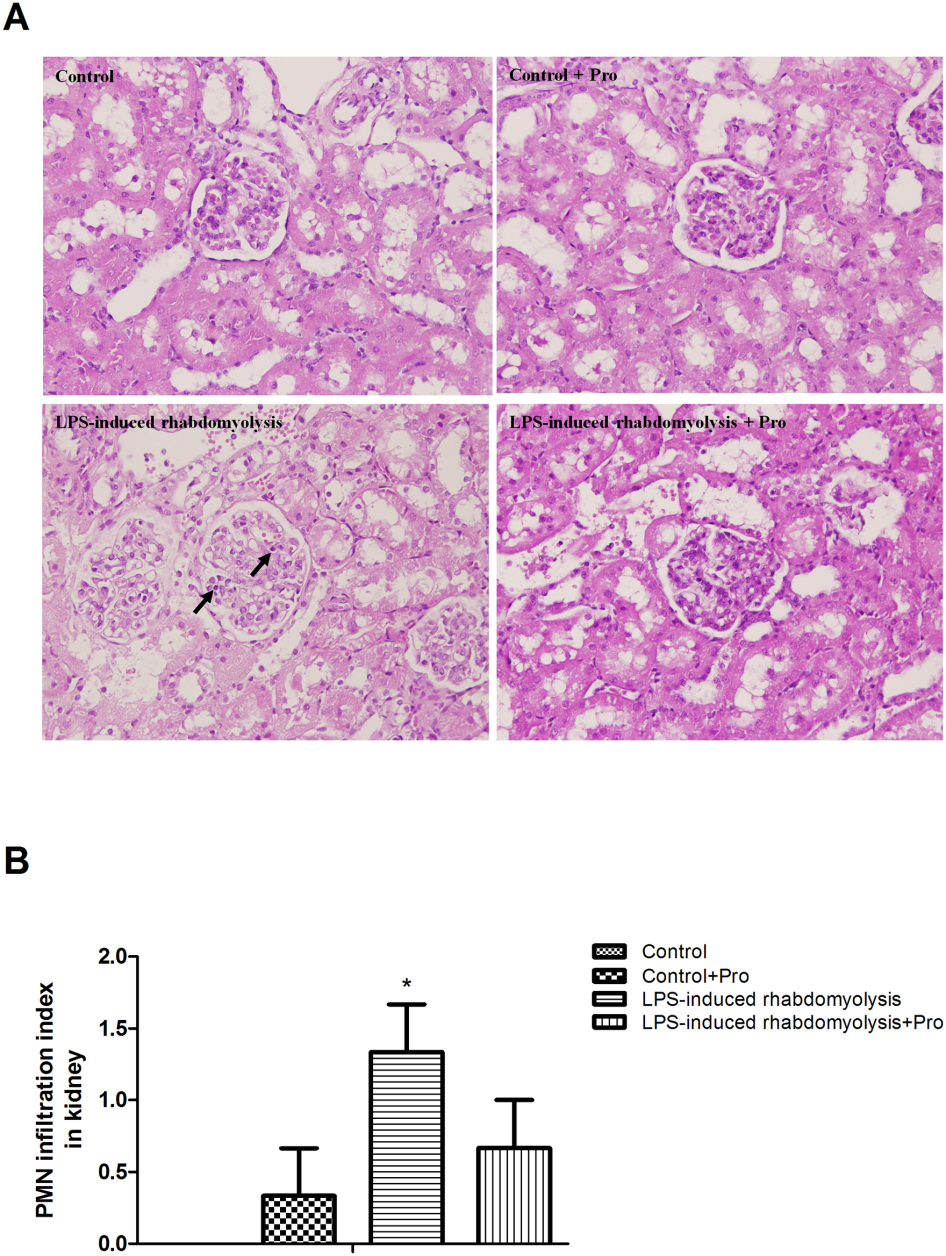
(A) Representative histopathologic features and (B) polymorphonuclear neutrophil (PMN) infiltration index of kidney tissue sections obtained from LPS-induced rhabdomyolysis rats. Depicted are changes in PMN infiltration index at the end of experiment (at 6 h) in different groups of animals that received vehicle at time 0 (Control, n = 3), received vehicle at time 0 and then received procainamide at 1 h (Control + Pro, n = 3), received LPS at time 0 (LPS-induced rhabdomyolysis, n = 3), and received LPS at time 0 and then received procainamide at 1 h (LPS-induced rhabdomyolysis + Pro, n = 3). All data are obtained using the same rats in Fig 1 and expressed as mean ± SEM. *P < 0.05, all versus Control rats; ^#^*P* < 0.05, with versus without procainamide in LPS-induced rhabdomyolysis rats. Arrows represent neutrophil infiltration. Original magnification x 400.

No significant changes in neutrophil infiltration were observed in the kidneys of Control and Control + Pro groups (Fig 6). The rats in the LPS-induced rhabdomyolysis group exhibited an overt neutrophil infiltration in the kidneys (Fig 6). Although LPS-induced rhabdomyolysis + Pro group had lower degree of neutrophil infiltration than LPS-induced rhabdomyolysis group, it was not significant (Fig 6).

### Effects of procainamide on plasma IL-6 levels

The baseline levels of plasma IL-6 were not significantly different between any of the groups (Table 2). The rats in the LPS-induced rhabdomyolysis group exhibited obvious rises in the levels of plasma IL-6 at 6 h (Table 2). The treatment of rhabdomyolysis animals with procainamide significantly attenuated the elevation of plasma IL-6 levels (Table 2). However, no significant changes in plasma IL-6 levels at 6 h were observed in the Control and Control + Pro groups (Table 2).

**Table 2:** Effects of procainamide on plasma IL-6 levels in LPS-induced rhabdomyolysis rats.

	0 h	6 h
**IL-6 (pg/mL)**		
Control	108 ± 4	111 ± 4
Control + Pro	111 ± 6	165 ± 15
LPS-induced rhabdomyolysis	116 ± 5	63431 ± 4641*
LPS-induced rhabdomyolysis + Pro	119 ± 7	26925 ± 6010*†

Shown are the changes in plasma levels of IL-6 in different groups of animals. Control, saline was given at time 0 (n = 6); Control + Pro, saline was given at time 0 and then 50 mg/kg procainamide was given at 1 h (n = 6); LPS-induced rhabdomyolysis, LPS was given at time 0 (n = 8); LPS-induced rhabdomyolysis + Pro, LPS was given at time 0 and then 50 mg/kg procainamide was given at 1 h (n = 8). All data are expressed as mean ± SEM.

*P < 0.05, all versus Control rats;

^†^*P* < 0.05, with versus without procainamide in LPS-induced rhabdomyolysis rats.

### Effects of procainamide on the expression of DNMTs

**Fig 7:**
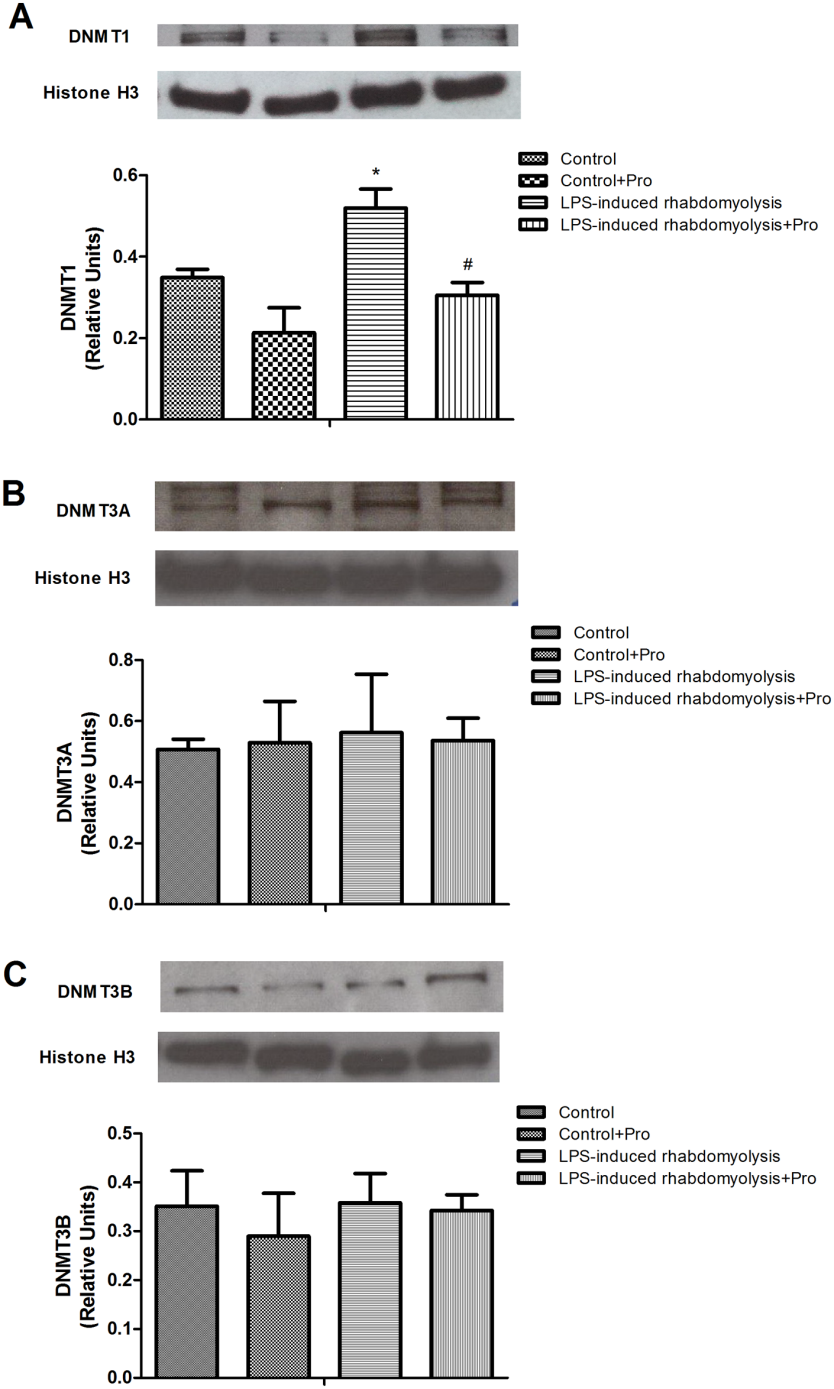
Effects of procainamide on the expressions of (A) DNMT1, (B) DNMT3A, and (C) DNMT3B in the lung of LPS-induced rhabdomyolysis rats. Depicted are changes in lung DNMT1, DNMT3A, and DNMT3B expressions at the end of experiment (at 6 h) in different groups of rats that received vehicle at time 0 (Control, n = 3), received vehicle at time 0 and then received procainamide at 1 h (Control + Pro, n = 3), received LPS at time 0 (LPS-induced rhabdomyolysis, n = 3), and received LPS at time 0 and then received procainamide at 1 h (LPS-induced rhabdomyolysis + Pro, n = 3). All data are obtained using the same rats in Fig 1 and expressed as mean ± SEM. *P < 0.05, all versus Control rats; ^#^*P* < 0.05, with versus without procainamide in LPS-induced rhabdomyolysis rats.

The expressions of DNMT1, DNMT3A, and DNMT3B were detectable in the lung homogenates obtained from the Control group (Fig 7A–7C). In the Control + Pro group, the levels of DNMT1, DNMT3A, and DNMT3B were similar to the Control group (Fig 7A–7C). The rats in the LPS-induced rhabdomyolysis group only exhibited significant elevations of DNMT1 levels in the lung (Fig 7A). Procainamide administration attenuated the increases of DNMT1 levels in the lungs of rats with rhabdomyolysis (Fig 7A). However, there was no significant difference in the expressions of DNMT3A and DNMT3B between the LPS-induced rhabdomyolysis and LPS-induced rhabdomyolysis + Pro groups (Fig 7B and 7C).

### Effects of hydralazine on hemodynamic variables, blood glucose, muscle injury, and renal function

The administration of rhabdomyolysis rats with hydralazine improved hypoglycemia, but had no effects on hypotension and tachycardia at 6 h after LPS (Table 3). In addition, the elevations of LDH and CRE in rhabdomyolysis rats were significantly reduced by hydralazine administration (Table 3). In the Control and Control + HYD groups, hemodynamics, blood glucose, LDH and CRE levels were not changed during the experimental period.

**Table 3:** Effects of DNMT inhibitor hydralazine on LPS-induced rhabdomyolysis rats.

	0 h	1 h	2 h	4 h	6 h
**MAP (mmHg)**					
Control	122 ± 7	123 ± 8	120 ± 8	118 ± 7	119 ± 7
Control + HYD	125 ± 8	112 ± 9	116 ± 8	118 ± 8	117 ± 7
LPS-induced rhabdomyolysis	122 ± 4	73 ± 3*	100 ± 3	100 ± 8	85 ± 9*
LPS-induced rhabdomyolysis + HYD	112 ± 3	66 ± 3*	87 ± 2*	88 ± 3*	87 ± 3*
**HR (beats/min)**					
Control	361 ± 17	367 ± 17	353 ± 10	370 ± 17	367 ± 20
Control + HYD	359 ± 20	370 ± 24	381 ± 21	387 ± 19	402 ± 18
LPS-induced rhabdomyolysis	376 ± 12	405 ± 22	522 ± 23*	541 ± 19*	537 ± 21*
LPS-induced rhabdomyolysis + HYD	355 ± 10	446 ± 27	494 ± 16*	514 ± 12*	528 ± 9*
**Blood glucose (mg/dL)**					
Control	103 ± 4	98 ± 3	108 ± 4	105 ± 4	112 ± 6
Control + HYD	106 ± 5	107 ± 6	106 ± 5	100 ± 5	103 ± 5
LPS-induced rhabdomyolysis	100 ± 3	230 ± 12*	98 ± 4	82 ± 8	55 ± 12*
LPS-induced rhabdomyolysis + HYD	96 ± 4	251 ± 14*	107 ± 16	89 ± 4	86 ± 2†
**LDH (U/L)**					
Control	158 ± 27	127 ± 23	122 ± 19	104 ± 25	101 ± 13
Control + HYD	275 ± 11	167 ± 10	158 ± 13	133 ± 18	150 ± 10
LPS-induced rhabdomyolysis	199 ± 47	336 ± 54	488 ± 97	1845 ± 742	6378 ± 2946*
LPS-induced rhabdomyolysis + HYD	140 ± 11	197 ± 15	291 ± 43	972 ± 440	1633 ± 1053†
**CRE (mg/dL)**					
Control	0.20 ± 0	0.20 ± 0	0.20 ± 0	0.20 ± 0	0.20 ± 0
Control + HYD	0.20 ± 0	0.20 ± 0	0.20 ± 0	0.20 ± 0	0.20 ± 0
LPS-induced rhabdomyolysis	0.20 ± 0	0.26 ± 0.02	0.30 ± 0.05*	0.40 ± 0.03*	0.52 ± 0.04*
LPS-induced rhabdomyolysis + HYD	0.20 ± 0	0.26 ± 0.04	0.22 ± 0.02†	0.22 ± 0.02†	0.24 ± 0.02†

Shown are the changes in MAP, HR, blood glucose, LDH, and CRE during the experimental period in different groups of animals. Control, saline was given at time 0 (n = 5); Control + HYD, saline was given at time 0 and then 0.3 mg/kg hydralazine was given at 0.5 h (n = 5); LPS-induced rhabdomyolysis, LPS was given at time 0 (n = 5); LPS-induced rhabdomyolysis + HYD, LPS was given at time 0 and then 0.3 mg/kg hydralazine was given at 0.5 h (n = 5). All data are expressed as mean ± SEM.

*P < 0.05, all versus Control rats;

^†^*P* < 0.05, with versus without hydralazine in LPS-induced rhabdomyolysis rats.

## Discussion

The clinical syndromes and complications of rhabdomyolysis such as high plasma CK levels, acute kidney injury, hyperkalemia, hypocalcemia, and acidosis were also observed in LPS-induced rhabdomyolysis animals. In addition, hypotension, tachycardia, and hypoglycemia occurred at the late stage of rhabdomyolysis induced by LPS. The exposure of rats to LPS increased the expression of lung DNMT1 and resulted in the elevation of plasma IL-6 levels. Treatment of LPS-induced rhabdomyolysis rats with procainamide improved muscle injury, renal dysfunction, electrolytes imbalance, metabolic acidosis, hypotension, and hypoglycemia. In addition, another DNMT inhibitor hydralazine diminished hypoglycemia, muscle damage, and renal dysfunction in rhabdomyolysis rats. Thus, therapeutic effects of procainamide could be attributed to the attenuation of DNMT1 overexpression and IL-6 overproduction.

The status of DNA methylation in the host is altered after infection with the pathogenic bacteria [24]. Previous studies exhibit that uropathogenic *Escherichia coli* can increase the expressions of DNMT1 and the levels of DNA methylation in uroepithelial cells [11, 12]. In addition, LPS is able to augment the production of inflammatory cytokines through the enhancement of DNMTs and DNA methylation [13, 14]. Aberrant DNA methylation of rat lungs is involved in the pathophysiology of LPS-induced acute lung injury [25]. Our study showed that LPS up-regulated the expression of DNMT1 in the lung and increased the level of IL-6 in the plasma. Overt DNA methylation and IL-6 production were significantly improved after treatment of LPS-induced rhabdomyolysis rats with DNMT1 inhibitor procainamide, indicating a crucial role of DNA methylation in the accumulation of pro-inflammatory cytokines. It has been demonstrated the initiation and progression of inflammation are associated with DNA methylation status in some inflammatory diseases [16, 26]. These findings suggest that procainamide could be a promising drug to diminish inflammatory response by reversing abnormal DNA methylation.

The association between pro-inflammatory cytokines and muscle damage has been reported [27, 28]. It is suggested that IL-6 may initiate the breakdown of skeletal muscle [29]. Rapid destruction of skeletal muscle integrity and leakage of intracellular contents (i.e. CK and LDH) into the extracellular space could lead to rhabdomyolysis. Plasma CK is usually regarded as the most effective biomarker in patients with rhabdomyolysis [30]. In the present study, the levels of IL-6 were increased in the plasma of animals treated with LPS, and this increment of IL-6 may result in the elevations of plasma CK and LDH concentration. Because kidney has been suggested to be the route for CK elimination, the influence of renal function on plasma CK level could not be excluded. The decrease of plasma CK level at 4 h in rhabdomyolysis group could be attributed to a sufficient compensation of CK excretion via the kidney. However, the severe renal impairment at 6 h after LPS could lead to the increase of plasma CK level. The increases of plasma IL-6, CK, and LDH levels in rats treated with LPS were suppressed by procainamide. This is in accordance with some previous works, showing the anti-inflammatory effects of DNMT inhibitors on macrophage and endothelial inflammation [16, 17]. Consequently, the attenuation of muscle injury by procainamide could be attributed to the reduction of IL-6 levels in the plasma of rats. In addition, release of numerous organic acids from damaged or hypoxic muscle cells provokes metabolic acidosis [31]. Acidosis has detrimental effects on various metabolic functions and would intensify the hyperkalemia. In the present study, the decreases of HCO_3_^-^ and BE levels in the arterial blood as well as hyperkalemia were observed in rats with rhabdomyolysis caused by LPS. Thus, metabolic acidosis and hyperkalemia were relieved after treating rhabdomyolysis rats with procainamide, indicating that procainamide has beneficial effects on the mitigation of plasma IL-6 levels and muscle injury. Moreover, metabolic acidosis would lower urinary pH and enhance the deposition of myoglobin and uric acid in the kidney, further exacerbating the renal function. The improvement of metabolic acidosis by procainamide could be an important contributor to the amelioration of acute kidney injury.

Indeed, one potential complication of severe rhabdomyolysis is acute kidney injury. The outcome of rhabdomyolysis is usually worse if renal damage develops [1]. Serum creatinine level is the most common marker of renal function in hospital. Recent clinical evidence suggests that poor patient survival in rhabdomyolysis is related to the concentration of plasma creatinine and the progression of acute kidney injury [8]. Attenuating acute kidney injury is the most important treatment strategy in patients with rhabdomyolysis. In our study, rats with rhabdomyolysis induced by LPS exhibited significant elevations of creatinine in the plasma and PMN infiltration in the kidney, indicating the development of renal dysfunction as seen in clinical patients with severe rhabdomyolysis. However, treatment of rhabdomyolysis rats with procainamide not only reduced the deterioration of renal function but also alleviated renal inflammation partially. This suggests that procainamide treatment protects rats against acute kidney injury resulting from rhabdomyolysis. In addition, the pathophysiology of renal toxicity induced by rhabdomyolysis is due to the deleterious effects of reactive oxygen species [32]. We found that superoxide levels were significantly increased in the kidneys of animals with rhabdomyolysis, and administration of procainamide ameliorated this overproduction. Growing evidence shows that oxidative stress is associated with the changes in DNA methylation status. Excessive DNA methylation of antioxidant genes contributes to the pathogenesis of oxidative stress in some diseases [33, 34]. Thus, DNMT inhibitors such as procainamide could improve acute kidney injury in rhabdomyolysis through its antioxidant effects.

Electrolyte disorders, such as hyperkalemia and hypocalcemia, are other major complications of rhabdomyolysis [35]. The release of large amounts of potassium and phosphate from destroyed muscle cells is one of the possibilities to induce hyperkalemia and hyperphosphatemia in rhabdomyolysis. Acute kidney injury can further aggravates hyperkalemia and hyperphosphatemia owing to decreasing urinary potassium and phosphate excretion [3]. High levels of phosphate trigger the deposition of calcium phosphate, contributing to the hypocalcemia. Similarly, our LPS-induced rhabdomyolysis rats also displayed hyperkalemia and hypocalcemia. After treatment of rhabdomyolysis rats with procainamide, marked improvement in hyperkalemia was observed. Procainamide alleviated the elevation of plasma potassium levels probably by mitigating muscle damage and acute kidney injury in rhabdomyolysis animals. However, procainamide exhibited less therapeutic effects on hypocalcemia in rhabdomyolysis rats induced by LPS. This implies that severe hypocalcemia needs to be further managed even when procainamide is used for the treatment of rhabdomyolysis.

In our animal model, the rats not only showed typical complications of rhabdomyolysis but also displayed some systemic manifestations, such as hypotension and hypoglycemia. This may be due to injured muscle cells markedly increase the membrane permeability, causing shifts of sodium and water from the intravascular to interstitial compartments. This fluid shift may lead to intravascular hypovolemia and hemodynamic instability [36]. Furthermore, LPS causes the expression of inducible nitric oxide synthase, eliciting nitric oxide overproduction, and hence, hypotension in the animals [37]. These may result in hypotension as seen in this study. In addition, the rhabdomyolysis patient with carnitine palmitoyltransferase deficiency exhibits severe hypoglycemia [38]. LPS reduces expressions of major proteins for fatty acid oxidation, including carnitine palmitoyltransferase [39]. Thus, exposure to LPS would inhibit glucose production and provoke hypoglycemia [40]. Our data revealed that procainamide administration significantly alleviated the hypotension and hypoglycemia in LPS-induced rhabdomyolysis rats. Despite procainamide seemed to be effective in the treatment of LPS-induced hypotension and hypoglycemia in this study, we need more studies to unveil the mechanisms of this drug.

Our results provide *in vivo* evidence that LPS could induce DNMT1 overexpression and IL-6 overproduction resulting in clinical manifestations of rhabdomyolysis which can be improved with procainamide treatment. Furthermore, similar therapeutic effects on hypoglycemia, muscle damage, and renal dysfunction were observed in rhabdomyolysis rats treated with another DNMT inhibitor (e.g. hydralazine). These findings reveal a crucial role for DNA methylation in the progression and regulation of endotoxin-induced rhabdomyolysis. Therefore, we suggest that DNMT1 inhibitors (e.g. procainamide) could be promising drugs for ameliorating serious complications in LPS-induced rhabdomyolysis.
